# A splice site mutation in the *PAX6* gene which induces exon skipping causes autosomal dominant inherited aniridia

**Published:** 2012-03-29

**Authors:** Nicole Weisschuh, Bernd Wissinger, Eugen Gramer

**Affiliations:** 1 Centre for Ophthalmology, Institute for Ophthalmic Research, Molecular Genetics Laboratory, Tuebingen, Germany; 2 University Eye Hospital, Julius-Maximilians-University, Wuerzburg, Germany

## Abstract

**Purpose:**

To identify the underlying genetic cause in a two generation German family diagnosed with isolated aniridia.

**Methods:**

All patients underwent full ophthalmic examination. Mutation screening of the paired box gene 6 (*PAX6*) was performed by bidirectional Sanger sequencing. A minigene assay was applied to analyze transcript processing of mutant and wildtype *PAX6* variants in HEK293 cells.

**Results:**

We identified a *PAX6* sequence variant at the splice donor site (+5) of intron 12. This variant has been described before in another family with aniridia but has not been characterized at the transcript level. We could demonstrate that the mutant allele causes the skipping of exon 12 during transcript processing. The mutation is predicted to result in a ‘run on’ translation past the normal translational stop codon.

**Conclusions:**

A splice site mutation resulting in exon skipping was found in a family with autosomal dominant aniridia. The mutation is predicted to result in an enlarged protein with an extra COOH-terminal domain. This very likely affects the transactivation properties of the PAX6 protein.

## Introduction

Aniridia is a rare, bilateral, congenital ocular disorder causing incomplete formation of the iris. The degree of iris hypoplasia is variable, ranging from minimal loss of iris tissue to nearly complete absence. The visual impairment caused by iris hypoplasia might be enhanced by several ocular complications, including cataract, glaucoma and corneal clouding. Aniridia can be associated with extra-ocular anomalies, including Wilms tumor, genitourinary anomalies and mental retardation (OMIM 194072); absent patella (OMIM 106220); and cerebellar ataxia and mental retardation (OMIM 206700). In isolated aniridia (OMIM 106210), familial cases are relatively frequent and characterized by an autosomal dominant mode of inheritance [1]. Most, if not all cases with isolated aniridia, can be attributed to mutations in the paired box gene 6 (*PAX6*) [2]. PAX6 is a member of the PAX multigene family of transcription factors that are involved in embryonic differentiation [3,4].

Numerous *PAX6* mutations have been detected in patients with aniridia. A compilation of these mutations can be found in the Human PAX6 Mutation Database [5]. Most of mutations are nonsense mutations, splice site mutations or insertion/deletions which are predicted to result in premature termination codons. The remaining mutations are missense or run-on mutations [6].

The aim of the present study was to identify the underlying genetic cause in a Germany family with autosomal dominant aniridia.

## Methods

### Patients

Four family members in two successive generations were diagnosed with apparently isolated aniridia at the University Eye Hospital in Wuerzburg, Germany. Ophthalmic examination included best corrected visual acuity, measurement of intraocular pressure, slit lamp examination, and funduscopy.

Informed consent, conform to the Institutional Review Board requirements, were obtained from two individuals to participate in the molecular genetic study. Genomic DNA was extracted from peripheral blood leukocytes using standard protocols.

### Mutation screening

Mutation screening of the *PAX6* gene (RefSeq: NM_000280.3) was performed by bidirectional Sanger sequencing. Gene specific PCR primers were designed and used to amplify individual exons and flanking intron sequences applying standard PCR amplification protocols. Primer sequences are given in Table 1. PCR fragments were purified by ExoSAP-IT treatment (USB, Cleveland, OH), sequenced using Big Dye Termination chemistry (Applied Biosystems [ABI], Weiterstadt, Germany) and products separated on a DNA capillary sequencer (ABI 3100 genetic analyzer; ABI, Weiterstadt, Germany).

**Table 1:** Primer sequences for PCR amplification of *PAX6.*

Primer name	Primer sequence	Annealing temperature (°C)	Amplicon size (bp)
PAX6-Exon4-f	tgtaggggaaacaga	55	491
PAX6-Exon4-r	atcgagaagagccaagcaaa		
PAX6-Exon5-f	ctggtggtcctgttgtcctt	55	446
PAX6-Exon5-r	atgaagagagggcgttgaga		
PAX6-Exon6-f	gggctacaaatgtaattttaagaa	55	509
PAX6-Exon6-r	agagagggtgggaggaggta		
PAX6-Exon7-f	gagctgagatgggtgactg	55	300
PAX6-Exon7-r	gagagtaggggacaggcaaa		
PAX6-Exon8-f	tcaggtaactaacatcgca	55	719
PAX6-Exon8-r	gttgactgtacttggaagaa		
PAX6-Exon9-f	aggtgggaaccagtttgatg	55	311
PAX6-Exon9-r	catggcagcagagcatttag		
PAX6-Exon10-f	gctaaatgctctgctgccat	55	329
PAX6-Exon10-r	agagtgagagtcagagcccg		
PAX6-Exon11-f	ccgggctctgactctcact	55	221
PAX6-Exon11-r	gccactcctcacttctctgg		
PAX6-Exon12-f	gaggcttgatacataggc	55	452
PAX6-Exon12-r	ccataagaccaggagatt		
PAX6-Exon13-f	tccatgtctgtttctcaaagg	55	220
PAX6-Exon13-r	tcaactgttgtgtccccatag		

### Heterologous splicing assay

A 662-bp genomic segment of *PAX6* covering exon 12 with adjacent intronic sequences was amplified from patient genomic DNA using specific primers carrying 5′tails with NotI and BamHI recognition sequences. Since the variant c.1183+5G>A was present in heterozygous state in our patients, both the normal and the variant allele could be co-amplified. The PCR fragment was then cloned into the pCR2.1 plasmid (Invitrogen-Life Technologies, Karlsruhe, Germany). Cloned inserts were sequenced to identify clones carrying the normal c.1183+5 G-allele or the mutant c.1183+5 A-allele. Wildtype and mutant inserts were excised by digestion with BamHI and NotI and cloned into BamHI/NotI– digested pSPL3_2096 (a derivative of the exon-trapping vector pSPL3 [Invitrogen-Life Technologies], with a stuffer fragment cloned into the original NotI site).

HEK 293 cells were cultured in DMEM medium supplemented with 10% FBS at 37 °C, in 5% CO_2_. Six-well plates were used and the cells were cultured up to 75% of confluence. Cells were transfected with pSPL3-*PAX6* constructs with transfection reagent (Lipofectamine; Invitrogen-Life Technologies) according to the manufacturer's instructions. As a control, cells were transfected with pSPL3 lacking the *PAX6* insert. Total RNA was extracted 24 h after transfection with the RNAEasy Mini Kit (Qiagen, Hilden, Germany). Reverse transcription was performed applying the SA2 primer (a reverse primer located on the 3′tat exon of the pSPL3 vector) and the Transcriptor High Fidelity cDNA Synthesis Kit (Roche Applied Science, Mannheim, Germany). The cDNA was PCR amplified with pSPL3 exon primers and sequenced as described above.

## Results

**Figure 1:**
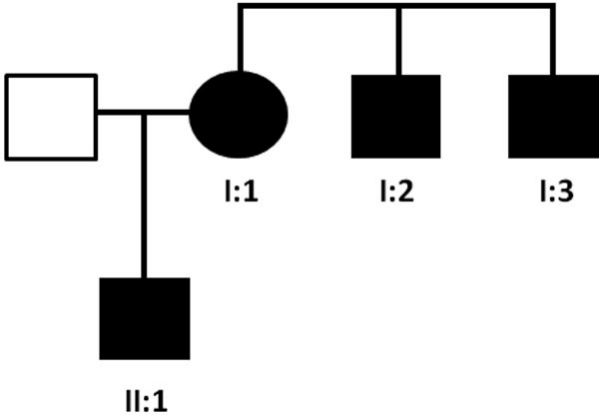
Pedigree of family. The family history revealed four affected members in two generations. Squares and circles symbolize males and females, respectively. Clear and blackened symbols denote unaffected and affected individuals. Patients I:1, I:2, I:3, and II:1 were clinically analyzed; blood samples for genetic analysis were available for patients I:1 and II:1.

**Figure 2:**
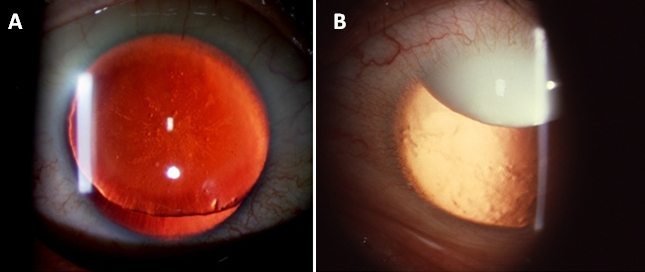
Retroillumination anterior segment photographs of affected family members. Proband II:1 shows total aniridia (**A**); proband I:2 shows subluxated cataractous lens (**B**).

Four family members from two successive generations of a German family underwent repeated ophthalmic examination. A diagnosis of isolated aniridia was made according to the fact that Wilm’s tumor and urogenital anomalies could be excluded in the patients and neurodevelopmental delay was not evident. However, a syndromic form of aniridia associated with brain abnormalities could not be excluded since magnetic resonance imaging was not performed. Follow-up ranged from four years (patient II:1) to 40 years (patient I:1). The pedigree of the family is given in Figure 1. Absent or nearly absent irides as well as upward lens dislocation were diagnosed in all four patients. Patients I:2 and I:3 also presented with congenital cataract. Ophthalmic findings are compared and summarized in Table 2 and selected clinical features of two probands are shown in Figure 2.

**Table 2:** Clinical features of probands.

**Patient**	**Aniridia**	**Lens subluxation**	**Congenital cataract**	**Congenital nystagmus**	**Secondary glaucoma**
I:1	+	+	-	-	+
I:2	+	+	+	+	+
I:3	+	+	+	-	+
II:1	+	+	-	-	-

**Figure 3:**
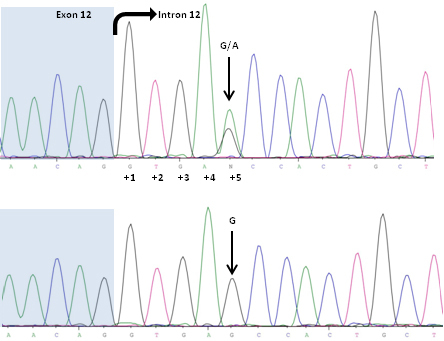
Forward sequence analysis of exon 12 (highlighted in blue) and adjacent intronic sequence of the *PAX6* gene in patient II:1 (upper panel) and a healthy control person (lower panel). The arrow indicates the heterozygous G>A transition in intron 12 at position +5.

**Figure 4:**
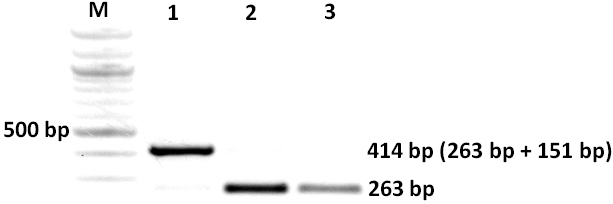
Agarose gel electrophoresis of RT–PCR products. RNA was derived from HEK293 cells transfected with the wildtype c.1183+5 G-allele plasmid construct (lane 1), the mutant c.1183+5 A-allele plasmid construct (lane 2) and the empty pSPL3 vector (lane 3). M, size ladder (100 bp ladder, Fermentas, St. Leon-Rot, Germany). The RT–PCR for the empty pSPL3 vector gives rise to a PCR product of 263 bp. Exon 12 of the PAX6 gene comprises 151 nucleotides.

**Figure 5:**
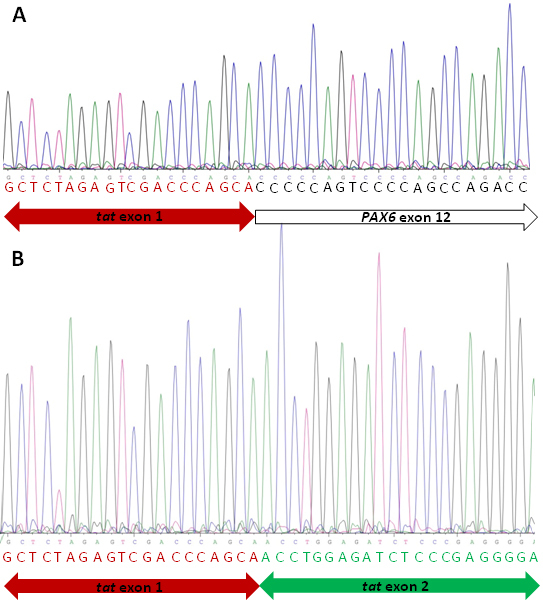
Sequence analysis of RT–PCR products. RNA was derived from HEK293 cells transfected with the wildtype c.1183+5 G-allele plasmid construct (**A**) and the mutant c.1183+5 A-allele plasmid construct (**B**). **A**: Red letters indicate the tat exon 1 sequence and black letters indicate sequence of *PAX6* exon 12. **B**: Red letters indicate the tat exon 1 sequence and green letters indicate the tat exon 2 sequence.

**Figure 6:**
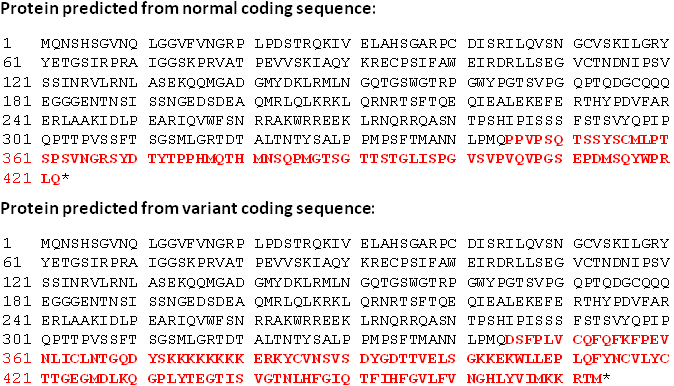
Predicted amino acid sequence of normal and mutant transcript. Deletion of nucleotides c.1033_1183 is predicted to replace the last 78 amino acids of the PAX6 protein with an enlarged peptide sequence of 129 amino acid residues.

Bidirectional sequencing of *PAX6* exons did not reveal any causative alteration but the analysis of exon/intron boundaries resulted in the identification of a nucleotide substitution at the splice donor sequence of intron 12 (Figure 3). Using online resources, we evaluated the presumptive effect of this variant on the splice donor site. The wild-type and mutant sequences (exon 12 plus approximately 250 bp of each of the adjacent introns) were submitted to three different splicing prediction tools: NNSPLICE [7], Geneid [8], and HSF [9]. As predicted by all three tools, the variant sequence substantially decreased the recognition of the intron 12 donor site (Table 3). We therefore reasoned that the c.1183+5G>A substitution affects splicing of *PAX6* and tested this substitution in a heterologous splicing assay. A genomic segment that included the complete exon 12 plus approximately 250 bp of each of the adjacent introns was amplified from patient II:1 and cloned into the exon trapping vector pSPL3. HEK 293 cells were transfected with plasmids carrying either the normal c.1183+5 G-allele or the mutant c.1183+5 A-allele, and RT–PCR products were amplified from RNA of transfected cell cultures with vector-specific primers. Cultures transfected with plasmid constructs with the mutant c.1183+5 A-allele yielded RT–PCR products that were clearly smaller than products from cultures transfected with the normal c.1183+5 G-allele (Figure 4), indicating abnormal splicing of the mutant minigene construct. Subsequent sequencing of the RT–PCR products showed that RT–PCR products derived from the transfection with the mutant c.1183+5 A-allele lacked exon 12 of the *PAX6* gene (Figure 5). As a result of this skipping of exon 12 which comprises 151 bp, the c.1183+5G>A variant is predicted to change the reading frame. Due to the lack of premature termination codons of this alternative reading frame, the deduced mutant polypeptide is larger than the wildtype PAX6 containing an extended COOH-terminal sequence (Figure 6).

**Table 3:** In silico prediction score values for the intron 12 donor splice site of the *PAX6* wildtype and mutant alleles.

**Splicing prediction tool**	Wildtype*	**Mutant**
NNSplice	1.00	0.74
Geneid	4.49	2.01
HSF	0.97	0.85

*The higher the value, the higher the probability of being a splice site in vivo.

## Discussion

Upon bidirectional Sanger sequencing of all coding exons and exon-intron boundaries of *PAX6*, we identified a sequence variant in the splice donor site of intron 12 (c.1183+5G>A). Although this variant has been described before in a small Swiss family (affected mother and son) with isolated aniridia [10], the pathogenic nature of the mutation has never been confirmed. Unlike the first and second positions of the splice donor sequence, the fifth is not invariant and the consequence of this change can only be tested by RNA analysis. To circumvent the lack of PAX6 expression in accessible tissues, we made use of a heterologous splicing assay to test in direct comparison mutant and wildtype *PAX6* minigene constructs. Human *PAX6* encodes a 422 amino acid transcription factor with two DNA-binding domains: a paired domain at the NH_2_-terminus and a homeodomain in the center of the protein. The COOH-terminal segment is rich in proline, serine, and threonine residues (PST domain) and constitutes the transactivating domain of the protein [11]. The PST domain is encoded by exons 10, 11, 12, and 13. The skipping of exon 12 as demonstrated in our family is predicted to result in a frameshift and ‘run on’ translation past the normal translational stop codon, thus generating an enlarged protein with an extra COOH-terminal domain. This very likely affects the transactivating domain of the protein. Skipping of exon 12 has also been demonstrated by Hanson and coworkers [12,13] in two aniridia families that had a deletion of the first 3 bp of intron 12 and a substitution of the last base of exon 12, respectively. Two other mutations in the splice donor site of intron 12 have been identified which probably also result in the out-of-frame skipping of exon 12 and a ´run on´ transcript, however, these have not been characterized on the RNA level [14,15]. These findings substantiate our hypothesis that the p.P345DfsX130 mutation is the underlying cause for the aniridia phenotype in our family. However, the functional significance of this mutation can only be assessed by in vivo transcriptional activation assays.

We observed a considerable phenotypic variability in our family (Table 1). Some features as upward lens dislocation could be seen in all members. The degree of iris hypoplasia was also strikingly similar. On the other hand, only two patients had congenital cataract and only one had nystagmus. PAX6 exerts its function through a complex pathway. Therefore it is not surprising that the phenotypic outcome of any particular mutation is dependent on an individual’s genetic background. In fact, phenotypic variability, either intra- or interfamilial, is often observed in aniridia [16-18]. Given that aniridia is a rare disease, genotype-phenotype correlations are difficult to assess. The most comprehensive analysis has been performed by Hingorani and colleagues [19]. The authors analyzed phenotypic data of 43 individuals diagnosed with aniridia or aniridia-like phenotypes and grouped them according to the type of mutation found in the *PAX6* gene (loss-of-function mutations, missense mutations, and COOH-terminal extension (CTE) mutations). They observed considerable phenotypic variability within each group but also noticed that individuals with missense mutations had the mildest phenotypes with better preserved iris structures. In their cohort of ten individuals with CTE-mutations, two were diagnosed with exudative retinopathy. More recently, Aggarwal and colleagues [15] reported a family with a mutation in the splice donor site of *PAX6* intron 12. Although not demonstrated experimentally, the mutation is predicted to result in CTE. The authors state that the presence of chorioretinal degeneration in one of the affected individual raises the possibility that CTE-mutations are associated with chorioretinal involvement in aniridia. We cannot substantiate this hypothesis. Chorioretinal involvement was excluded in our patients, except for patient I:2 in whom fundoscopy was compromised by nystagmus. Whether CTE alleles of PAX6 in fact provoke a distinct phenotypic outcome remains to be established and requires the analysis of further patients.
